# Treatment of Focal Chondral Lesions in the Patellofemoral Joint with Collagen Membrane: Clinical and Functional Outcomes in a Two-Year Follow-Up

**DOI:** 10.1055/s-0045-1809338

**Published:** 2025-06-23

**Authors:** Pedro Debieux, José Ricardo Dantas Moura Costa, Wesley Araujo Weis, Diego da Costa Astur, Camila Cohen Kaleka, Moisés Cohen

**Affiliations:** 1 Department of Orthopedic Surgery, Hospital Israelita Albert Einstein, São Paulo, SP, Brazil; 2 Department of Orthopedic Surgery, Hospital Beneficência Portuguesa de São Paulo, São Paulo, SP, Brazil; 3 Department of Orthopedic Surgery, Instituto Cohen, São Paulo, SP, Brazil; 4 Sports Traumatology Center, Department of Orthopedics and Traumatology, Escola Paulista de Medicina, Universidade Federal de São Paulo, São Paulo, SP, Brazil; 5 Department of Orthopedics, Empresa Cuiabana de Saúde Pública, Cuiabá, MT, Brazil

**Keywords:** cartilage, articular, chondrogenesis, knee injuries, cartilagem articular, condrogênese, traumatismos do joelho

## Abstract

**Objective:**

To evaluate the clinical and functional outcomes of patients undergoing surgical treatment to repair focal chondral lesions in the patella and trochlea using the autologous matrix-induced chondrogenesis (AMIC) technique after a minimum follow-up of 2 years.

**Methods:**

A total of 24 patients (25 knees) with a mean age of 39.6 ± 4.7 years underwent the patellofemoral AMIC technique and evaluation over a mean follow-up of 3.64 ± 0.65 years. We collected data on patient factors, lesion morphology, and patient-reported outcome measures, including the International Knee Documentation Committee (IKDC), Tegner, Kujala, Fulkerson, and Lysholm scores, as well as the Visual Analog Scale (VAS).

**Results:**

Male subjects accounted for 76% of the sample. The mean defect size of the chondral lesions was of 1.99 ± 0.36 cm ^2^ . All defects were of grade IV according to the Outerbridge classification. At the last follow-up, patients showed the following mean increases in the scores: Kujala – from 61.9 to 87.9; IKDC –from 51.3 to 83.6; Lysholm –from 64.0 to 88.4; Tegner –from 4.04 to 5.12; Fulkerson –from 60.2 to 89.3; and VAS – from 5.6 to 1.24. All results were statistically significant ( *p* < 0.05).

**Conclusion:**

The AMIC technique is a safe, effective, and feasible method to treat symptomatic full-thickness chondral defects of the patellofemoral cartilage in properly-selected cases, and it resulted in clinical and functional improvement in all criteria under analysis.

## Resumo

**Objetivo:**

Avaliar os desfechos clínicos e funcionais de pacientes submetidos a tratamento cirúrgico para reparo de lesão condral focal na patela e na tróclea, pela técnica de condrogênese induzida por matriz autóloga ( *autologous matrix-induced chondrogenesis* , AMIC, em inglês), após um mínimo de 2 anos de tratamento.

**Métodos:**

Ao todo, 24 pacientes (25 joelhos) com idade média de 39,6 ± 4,7 anos foram submetidos à técnica AMIC patelofemoral e foram avaliados em um seguimento médio de 3,64 ± 0,65 anos. Os fatores dos pacientes, a morfologia da lesão e as medidas de resultado relatadas pelos pacientes, incluindo os escores do International Knee Documentation Committee (IKDC), de Tegner, de Kujala, de Fulkerson, de Lysholm, e a Escala Visual Analógica (EVA), foram coletados.

**Resultados:**

O sexo masculino representou 76% da amostra. O tamanho médio do defeito das lesões condrais foi de 1,99 ± 0,36 cm ^2^ . Todos os defeitos foram classificados como de grau IV, de acordo com a classificação de Outerbridge. No seguimento final, os pacientes apresentaram os seguintes aumentos na pontuação média nos escores: Kujala – de 61,9 para 87,9; IKDC – de 51,3 para 83,6; Lysholm – de 64,0 para 88,4; Tegner – de 4,04 para 5,12; Fulkerson – de 60,2 para 89,3; e EVA – de 5,6 para 1,24. Todos os resultados apresentaram significância estatística ( *p* < 0,05).

**Conclusão:**

A AMIC é um método seguro, eficaz e viável para o tratamento de defeitos condrais sintomáticos, de espessura total da cartilagem femoropatelar, em casos adequadamente selecionados, e resultou em melhora clínica e funcional em todos os critérios analisados.

## Introduction

Articular cartilage is a highly specialized tissue whose main function is to enable multiplanar movement of the joint under several conditions of applied load. 1 Another significant role of hyaline cartilage is its ability to transmit load to the adjacent subchondral bone without damaging it. In addition, it provides a smooth, lubricated, low-friction surface that enables the maintenance of joint homeostasis. 2

Lesions affecting the patellofemoral region occur in up to one-third of the cases, and they can result in severely-limiting symptoms. 3 4 The most common causes include trauma, patellar dislocation, misalignment, and instability. 3 5 These lesions remain a significant challenge for orthopedic surgeons. 6 7 8 Several treatments have been proposed, with the recommendation of surgical therapy in cases in which conservative modalities have not been successful. The options available include microperforations, autologous chondrocyte implantation, and autologous osteochondral graft, but the ideal treatment remains controversial. 3 9

Autologous matrix-induced chondrogenesis (AMIC) is part of this technical arsenal to repair focal full-thickness cartilage lesions. This technique stimulates the bone marrow through microfractures and lesion covering with type-I and -III collagen membrane to contain the formed clot and promote adhesion, proliferation, and cell differentiation. 7 8 9 10 11 12 13 Notably effective in treating condylar chondral lesions, reports of outcomes from this technique on isolated patellofemoral compartment lesions are lacking. 9 14 However, since its introduction, AMIC has demonstrated good short- and medium-term outcomes. 7 8 10 15 16 17

Therefore, the present study aimed to analyze the clinical outcomes of patients undergoing surgical treatment to repair focal chondral lesions in the patella and trochlea using the AMIC technique with a follow-up of 2 years.

## Materials and Methods

The current is a descriptive and observational case series study. We obtained data by analyzing medical records of patients who underwent surgical treatment to repair focal chondral lesions in the patella or trochlea using the AMIC technique from 2015 to 2020. The institutional Ethics in Research Committee approved the study under number CAAE 65062522.9.0000.5505.

A single surgeon operated on 24 patients (25 knees), 19 men and 5 women, who were followed up for at least 2 years after the surgical procedure. All received detailed guidance on the proposed surgical technique and other available treatment options, with their respective advantages and disadvantages, and agreed with the procedure chosen.

The inclusion criteria were age from 15 to 60 years, diagnosis of focal chondral lesion in the patella or trochlea with an area ranging from 0.5 cm ^2^ to 4.0 cm ^2^ , International Cartilage Repair Society (ICRS) classification grade III or IV, regular physical activity, and lack of response to conservative treatment with physical therapy and functional rehabilitation for up to 6 weeks. The exclusion criteria were patellar malalignment, subchondral bone (osteochondral) lesions, advanced osteoarthritis (grade ≥ 2 on the Ahlbäck radiographic classification), and a history of cartilage repairs or concomitant procedures, such as meniscal repair or ligament reconstruction.

All patients with suspected patellar or trochlear chondral lesions underwent a preoperative evaluation, including radiographs and magnetic resonance imaging (MRI) scans to characterize and measure the chondral injury and identify potential ligament injuries or malalignments in the lower limbs. This approach enabled a careful selection of participants meeting the inclusion criteria.

## Surgical Technique

**Fig. 1:**
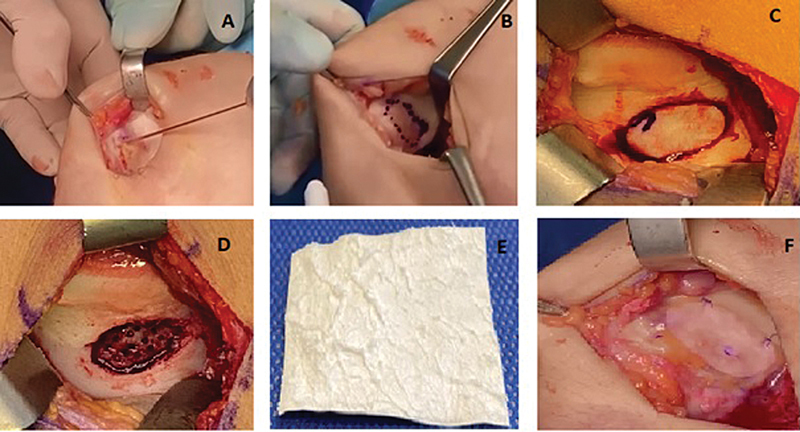
Clinical image of autologous matrix-induced chondrogenesis (AMIC) in a cartilage lesion. **A** , extensive cartilage defect. **B** , regularization of the lesion edges. **C** , marking of the lesion size after curettage. **D** , subchondral bone nanoperforation. **E** , porcine type I/III collagen membrane. **F** , final appearance of the membrane implant with 5-0 monofilamentous suture reinforced with fibrin glue.

The procedure begins with arthroscopy for a detailed evaluation of the chondral lesion and diagnostic confirmation, followed by medial or lateral parapatellar arthrotomy depending on the location of the lesion. After patellar eversion, the edges of the lesion are molded to create stable vertical walls of healthy adjacent cartilage ( Fig. 1 ). The defect is marked and curetted down to its calcified layer, followed by nanoperforation of the subchondral bone. Then, the porcine collagen type-I and -III membrane (Chondro-Gide, Geistlich Pharma AG) is applied over the lesion and fixed with 5–0 monofilament suture (PDS, Ethicon, Inc.), reinforced with fibrin glue (Tisseel, Baxter Medical Pharmaceutical Ltd.) at the edges ( Fig. 1 ).

## Rehabilitation

Postoperative rehabilitation includes initial immobilization with a hip brace for 10 days, with full weight bearing on the operated limb. The progressive gain in the range of motion starts according to pain tolerance, with ambulation without orthosis encouraged until gait normalization in 6 to 8 weeks. The patients are allowed to practice contact sports 6 to 8 months after surgery.

## Data Collection

We collected demographic data, such as sex, age, laterality, lesion location, size, follow-up, and associated procedures. The preoperative clinical evaluation used internationally-standardized clinical scores, including the Lysholm, Fulkerson, Kujala, Tegner, International Knee Document Committee (IKDC) scores, and the Visual Analogue Scale (VAS). These scores were applied again after 2 years of outpatient follow-up.

## Statistical Analysis

Data analysis used the following software: IBM SPSS Statistics for Windows, version 20.0 (IBM Corp.), Minitab 16 (Minitab, LLC), and Excel 2010 (Microsoft Corp.). The analysis of the quantitative variables was performed by calculating means, standard deviations (SDs), and quartiles. For the qualitative variables, absolute and relative frequencies were calculated. Result comparison between groups employed the Wilcoxon, Mann-Whitney, and equality of two proportions tests. Data was shown in tables and boxplots. The level of statistical significance was of 95%, and the *p* -value was set at 0.05.

## Results

We analyzed 24 patients (25 knees) aged 15 to 60 (mean: 39.6 ± 4.7) years, 5 women and 19 men. The lesions affected the patella in 12 cases and the femoral trochlea in 13 cases, and their sizes ranged from 1.0 cm ^2^ to 3.99 cm ^2^ in the patella and from 0.9 cm ^2^ to 2.94 cm ^2^ in the trochlea. The mean follow-up was of 3.54 ± 0.65 years. Table 1 shows the frequencies of the lesion site, laterality, and sex.

**Table 1:** Comparison between groups regarding the distribution of qualitative factors

		Patella		Trochlea		Total	
		N	%	N	%	N	%
Side	Right	7	58.3%	5	38.5%	12	48.0%
	Left	5	41.7%	8	61.5%	13	52.0%
Sex	Female	5	41.7%	1	7.7%	6	24.0%
	Male	7	58.3%	12	92.3%	19	76.0%
Site	Lateral	5	41.7%	5	38.5%	10	40.0%
	Medial	4	33.3%	3	23.1%	7	28.0%
	Medial and lateral	3	25.0%	5	38.5%	8	32.0%

The evaluation of the Lysholm, Fulkerson, Kujala, Tegner, VAS, and IKDC scores was performed before and after surgery, with a minimum follow-up of 24 months. There was a statistically significant increase in virtually all comparisons, except for the Tegner score for the trochlea group, which presented no statistical significance, even though the mean value increased from 4.46 to 5.31 ( *p* = 0.305). Table 2 compares the mean values for the patella and trochlea groups.

**Table 2:** Score comparison per group

			Mean	Median	Standard deviation	N	*p* -value
Lysholm	Patella	Pre	60.1	62.5	± 13.6	12	0.003
		Post	88.3	90.5	± 10.7	12	
	Trochlea	Pre	67.5	63.0	± 13.2	13	0.007
		Post	88.5	89.0	± 13.4	13	
IKDC	Patella	Pre	48.7	46.6	± 14.9	12	0.002
		Post	82.6	89.1	± 13.9	12	
	Trochlea	Pre	53.6	52.9	± 11.5	13	0.003
		Post	84.6	92.0	± 14.5	13	
Tegner	Patella	Pre	3.58	3.00	± 1.62	12	0.017
		Post	4.92	5.50	± 1.31	12	
	Trochlea	Pre	4.46	5.00	± 1.76	13	0.305
		Post	5.31	5.00	± 1.93	13	
Fulkerson	Patella	Pre	56.9	60.5	± 15.0	12	0.002
		Post	88.2	90.0	± 11.6	12	
	Trochlea	Pre	63.3	64.0	± 15.1	13	0.005
		Post	90.3	94.0	± 14.4	13	
VAS	Patella	Pre	5.83	6.50	± 2.48	12	0.005
		Post	1.42	0.50	± 1.93	12	
	Trochlea	Pre	5.38	6.00	± 2.06	13	0.003
		Post	1.08	0.00	± 1.71	13	
Kujala	Patella	Pre	58.3	58.5	± 14.5	12	0.002
		Post	86.4	89.0	± 12.9	12	
	Trochlea	Pre	65.2	70.0	± 14.1	13	0.006
		Post	89.2	91.0	± 12.8	13	

**Abbreviations:** IKDC, International Knee Documentation Committee; Post, postoperative period; Pre, preoperative period; VAS, Visual Analog Scale.

**Fig. 2:**
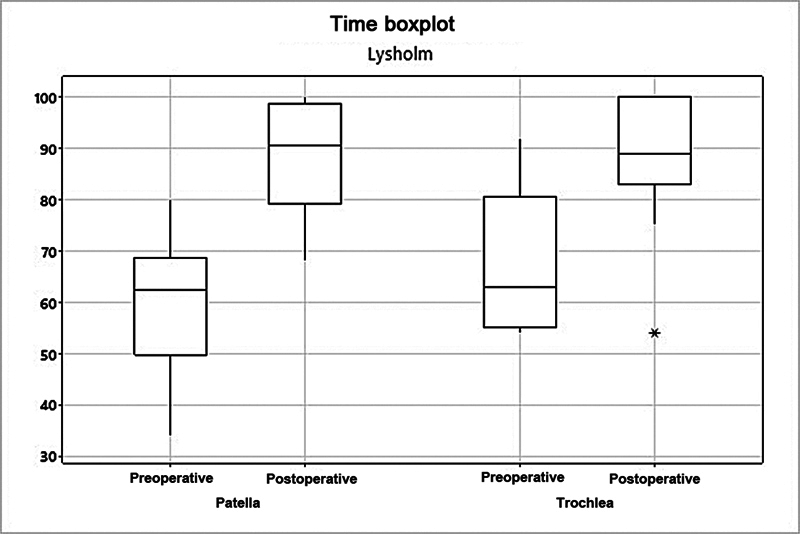
Analysis of the patella and trochlea groups per the Lysholm score.

**Fig. 3:**
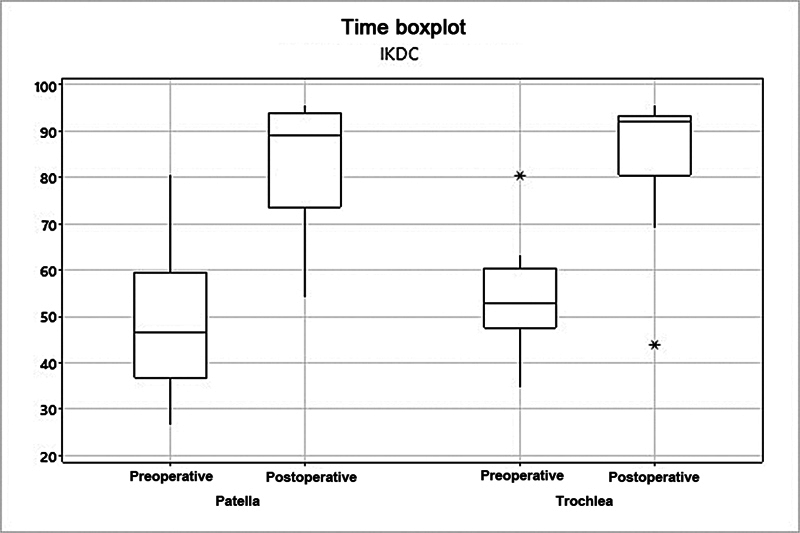
Analysis of the patella and trochlea groups per the International Knee Documentation Committee (IKDC) score.

**Fig. 4:**
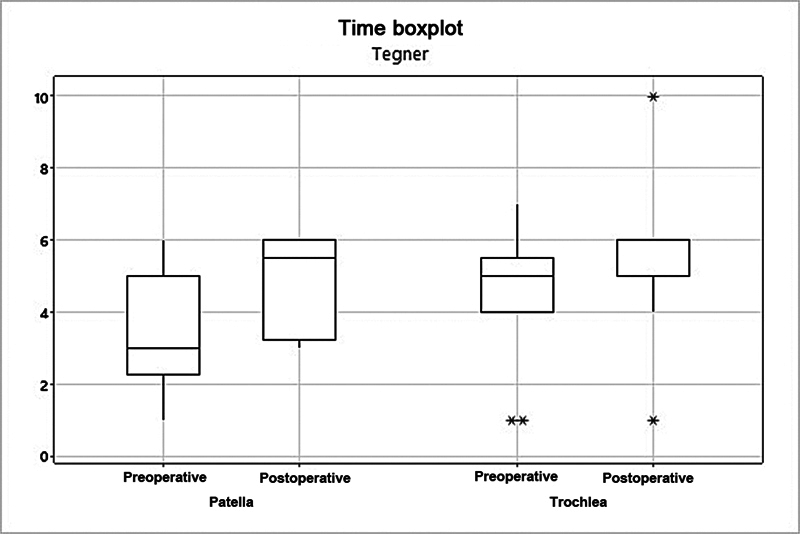
Analysis of the patella and trochlea groups per the Tegner score.

The mean Lysholm score increased from 60.1 to 88.3 ( *p* = 0.003) in the patella group, and from 67.5 to 88.5 ( *p* = 0.007) in the trochlea group ( Fig. 2 ). The mean IKDC score increased from 48.7 to 82.6 ( *p* = 0.002) in the patella group, and from 53.6 to 84.6 ( *p* = 0.003) in the trochlea group ( Fig. 3 ). The mean Tegner score increased from 3.58 to 4.92 ( *p* = 0.017) in the patella group ( Fig. 4 ).

**Fig. 5:**
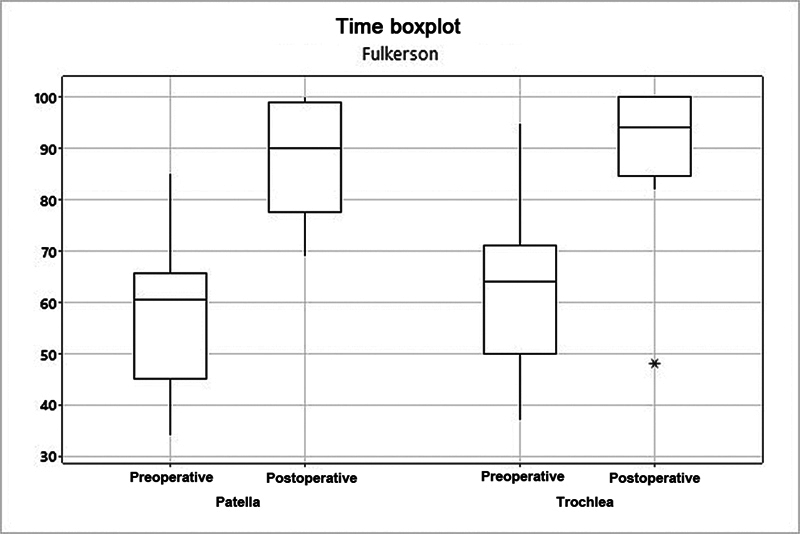
Analysis of the patella and trochlea groups at per Fulkerson score.

**Fig. 6:**
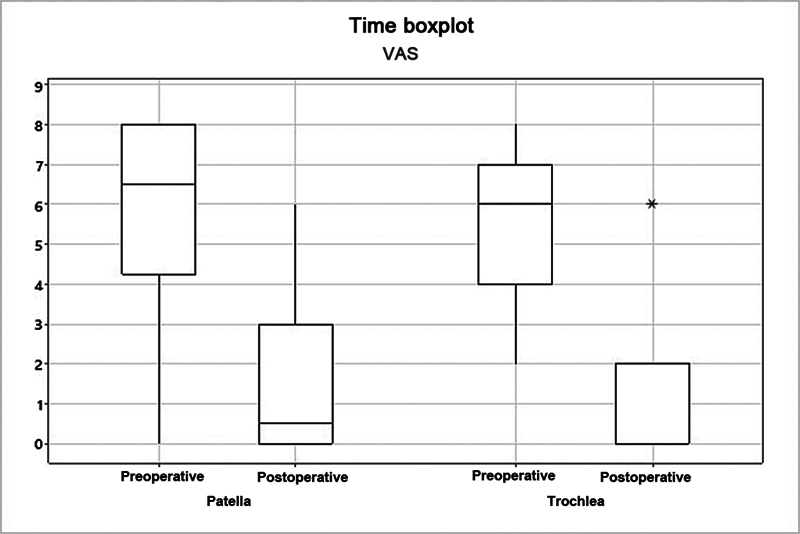
Analysis of the patella and trochlea groups per the score on the Visual Analog Scale (VAS).

**Fig. 7:**
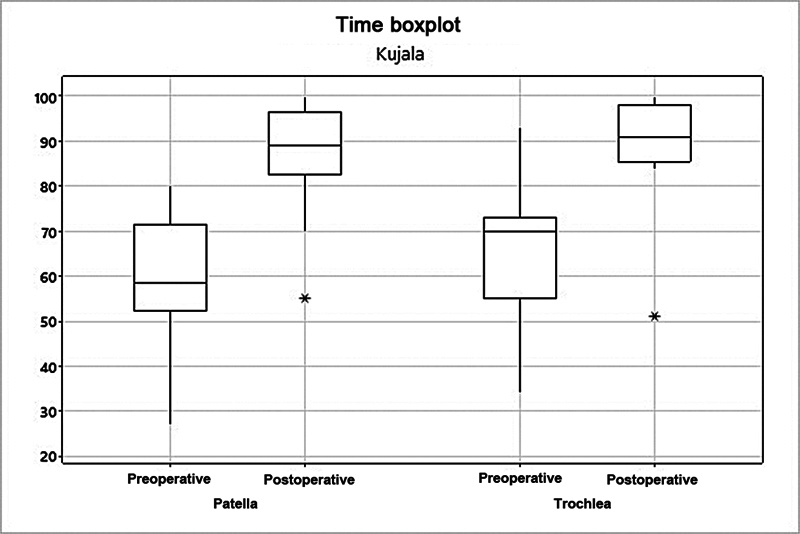
Analysis of the patella and trochlea groups per the Kujala score.

The mean Fulkerson score increased from 56.9 to 88.2 ( *p* = 0.002) in the patella group, and from 63.3 to 90.3 ( *p* = 0.005) in the trochlea group ( Fig. 5 ). The mean VAS score decreased from 5.83 to 1.42 ( *p* = 0.005) in the patella group, and from 5.38 to 1.08 ( *p* = 0.003) in the trochlea group ( Fig. 6 ). Lastly, the mean Kujala score increased from 58.3 to 86.4 ( *p* = 0.002) in the patella group, and from 65.2 to 89.2 ( *p* = 0.006) in the trochlea group ( Fig. 7 ).

## Discussion

The most relevant data from the current study was the efficacy of the AMIC technique to treat full-thickness chondral lesions in the patella and trochlea with a minimum follow-up of 2 years. All scores presented statistically significant clinical and functional improvements, resulting in symptom reduction and sports return at preinjury levels.

Consistent with these findings, Gille et al. 18 conducted a study with a sample of patients with patellofemoral lesions smaller than ours ( *n* = 11 versus *n* = 25), but a similar mean age. However, in their study, the patella was predominantly affected (nine versus two), while trochlear lesions were more frequent here (13 versus 12). Likewise, they 18 found improvements in Lysholm scores, which increased from 36 ± 21 preoperatively to 76 ± 24 at the 24-month follow-up. The Tegner and IKDC scores also showed a significant increase in patellar defects during the same analysis period, with these data published in a previous article. 10 Waltenspül et al. 3 observed an elevation in the Kujala score from 63.5 ± 11.6 preoperatively to 72.2 ± 17.4 after a minimum follow-up of 24 months ( *p* = 0.029).

Regarding patient satisfaction after AMIC, Panni et al., 19 after a mean follow-up of 7 years, reported that 76.2% of the patients considered the treatment good or excellent. Gille et al., 18 when evaluating 27 patients treated with AMIC, found that 87% of them were satisfied with the treatment outcomes. The current analysis confirms these data, revealing positive outcomes, with 84% of patients classifying their satisfaction as good or excellent, a rate consistent with those of the aforementioned literature.

In a 12-month follow-up, Tradati et al. 20 reported significant outcomes in patients undergoing AMIC. The Kujala score increased from 49.6 preoperatively to 87.8 after 12 months ( *p* < 0.01). The IKDC score increased from 36.1 to 79.8 ( *p* < 0.01). In addition, there was a significant reduction in pain, and the VAS score decreased from 7.5 to 1.5 ( *p* < 0.01). 20 The present study reveals that the benefits of the treatment proposed by Tradati et al. 20 are sustained for at least 24 months, since the variations in the scores measured here are symmetrical to those observed by those authors.

The patellofemoral compartment is the most challenging knee region to treat chondral lesions. Hinckel et al. 21 conducted a systematic review to establish which technique is most appropriate in this scenario. They observed that the outcomes of the combination of collagen membrane and autologous chondrocyte implant (ACI) were superior to those of the other techniques. Nevertheless, the AMIC technique was not included in their analysis 21 due to the lack of controlled studies at the time. The use of AMIC is well-established for the condylar region; Astur et al., 6 for instance, analyzed outcomes similar to those reported here in 15 patients for 12 months, noting a 4-point difference in VAS score at the end of the 12-month follow-up, consistent with our findings after 24 months, even though we dealt with the anterior knee compartment.

A current trend associates the classic AMIC technique herein described with orthobiological agents, preserving the subchondral bone or not. For instance, the AMIC plus technique associates bone marrow aspirate concentrate (BMAC) with the collagen membrane. This combination has shown evidence of postoperative pain relief, functional improvement, and outcome maintenance for up to 3 years. 22 Sciarretta et al. 23 reported promising results using the LIPO-AMIC technique, which combines AMIC with autologous adipose tissue grafting. These authors 23 observed early and progressive improvement, in addition to long-lasting symptom relief; moreover, they noted significant recovery and maintenance of daily functional and sports activities at 2-year and, especially, 5-year follow-ups. Another technique that deserves attention is minced cartilage implantation, which can be applied to small-diameter cartilage defects. This technique can be an alternative to bone marrow stimulation procedures, as minced cartilage implantation can be performed in a single-stage procedure, even using arthroscopic techniques. This minimally-invasive approach is easy to apply and an attractive option to treat focal chondral lesions. 24

The current study is not free from limitations, many of which are inherent to its design, as it is an observational case series study. First, the number of participants ( *n* = 25) may limit statistical robustness, despite being relevant compared with the international literature. Furthermore, the fact that it is a single-arm study and that does not include a control group prevents direct comparison and assessment of the relative efficacy of the interventions. Another limitation is the impossibility of comparing different techniques, such as nanofracture, since control groups are critical for data analysis. The heterogeneity of the lesions among the patients constitutes a significant limitation of the current study, since we included injuries in the trochlea and the patella. Moreover, we did not consider subtle variations in the anatomical and biomechanical patterns of the patellofemoral region. Another significant limitation is the lack of outcome assessment through imaging. Additionally, a longer follow-up could provide more robust data and enrich the conclusions.

## Conclusion

The repair of chondral lesions using collagen membrane is a safe, effective, and viable technique for the treatment of symptomatic full-thickness chondral defects of the patellofemoral cartilage in properly-selected cases, resulting in clinical and functional improvements in all criteria analyzed after 2 years of follow-up.
